# The lived experiences of resilience among Syrian refugees in the UK: interpretative phenomenological analysis

**DOI:** 10.1192/bjb.2022.16

**Published:** 2023-06

**Authors:** Mustafa Alachkar

**Affiliations:** Pennine Care NHS Foundation Trust, Ashton-under-Lyne, UK

**Keywords:** Refugees, mental health, resilience, Syria, stigma and discrimination

## Abstract

**Aims and method:**

Refugees’ mental health has attracted great interest from researchers recently, in view of increasing numbers of refugees settling in Europe. A deficit model, focusing on mental disorder, has often dominated the discourse on the subject, but a strength-based model is becoming more recognised and adopted. Through semi-structured interviews, and using interpretative phenomenological analysis as a data analysis tool, the current study sought to explore the lived experiences of Syrian refugees in the UK in relation to resilience factors.

**Results:**

Three main themes were identified reflecting interpersonal and family factors, factors related to religion, faith and belief systems, and personal qualities.

**Clinical implications:**

The study calls for perceiving refugees as resilient individuals with strengths and adaptive qualities. It also demonstrates that refugees’ resilience is essentially an interpersonal process, advocating therefore for engagement and therapeutic approaches that are systemic, relational, and culturally and spiritually competent.

Since the start of the popular uprising in Syria in 2011, the country has been going through political unrest that descended into a devastating war claiming the lives of several hundred thousand people and leaving many more injured or disappeared.^1^ The war has also led to the worst humanitarian and refugee crisis since the Second World War:^2,3^ over half of the Syrian population have been internally displaced (about 6.7 million) or have become refugees (about 6.6 million),^4^ and many of the latter have sought asylum in European countries, including the UK.

Refugees’ mental health has received increasing attention from researchers, along with increased awareness of the impact of trauma and stress on the psychological well-being of refugees.^5^ Researchers have often perceived refugees as traumatised and vulnerable victims, dependent on services and in need of support,^6–8^ with mental illnesses such as depression and post-traumatic stress disorder (PTSD) often seen as their default state.^9–11^ This approach may be understandable, given the high prevalence of mental health difficulties in this population^12,13^ and the multitude of traumatic events that many refugees experience,^13,14^ along with the difficulty in accessing help for their mental health for various reasons, including unstable living conditions, language barriers and stigma.^15^

Some researchers, however, assert that being a refugee is not in itself a psychological problem^10^ and that the consequences of refugees’ adverse experiences are better perceived as a normal response to their uncertain and challenging situation rather than a manifestation of psychopathology.^6^ Furthermore, responses to trauma that might seem pathological, such as PTSD, may be necessary and adaptive.^16^

This approach therefore prefers to see refugees as active survivors with agency and innate tendency towards healing and growth.^17^ In fact, by virtue of the fact that they have been able to survive the journey to a different country, adapt to a completely new culture and traditions, and learn a new language, refugees can be assumed to possess a characteristic level of resilience that is worth appreciating and exploring further.^18^ This is also supported by research suggesting that suicide rates among refugees settled in Western countries may be lower than rates among the ‘host population’.^19^

## Resilience

At the centre of this strength-based approach is the concept of resilience,^13^ a concept that is not easy to define although most people can readily relate to it. Resilience has been defined as the ability to ‘bounce back’ and move on in the face of trauma,^20^ or as ‘patterns of positive adaptation in the context of significant risk or adversity’ (p. 4)^21^ or as ‘the ability […] to maintain relatively stable, healthy levels of psychological and physical functioning’.^22^

Resilience has been linked to other concepts, such as coping, adaptation, recovery and post-traumatic growth.^23^ Exploring the relationship between resilience and these other concepts or between resilience and mental disorder is beyond the scope of this paper. It is worth noting, however, that although resilience may protect against the negative impact of adversity on mental health,^24,25^ having a mental disorder does not necessarily imply lack of resilience.^23^ Resilience is therefore best seen as a relational, contextual and culturally determined process that transcends psychiatric symptomatology and encompasses various personal and environmental qualities;^6^ furthermore, an individual may be resilient in some areas of functioning but not in others.^23^

Several research studies have looked at factors that aid refugees’ resilience. Among these are the role of family and social support, religion or spirituality, certain personal qualities, attitudes and belief systems and the meaning that refugees attribute to their experience.^8,22,26–30^

The resilience of Syrian refugees in Europe or the UK remains an insufficiently studied subject. Some studies have addressed resilience and related concepts, such as coping and post-traumatic growth among Syrian refugees in neighbouring countries. However, this has largely been through quantitative methods and questionnaires addressing coping, resilience and mental health.^31,32^

In their uniquely interesting study referred to above,^32^ Woltin et al compared Syrian refugees in Turkey with those in Germany in relation to regulatory focus and coping strategies and how these related to symptoms of mental illness. Differences were found between the two groups (refugees in Turkey were found to have higher levels of anxiety, depression and maladaptive coping). Importantly, and relevant to the current study, the study found that an individual's personality and regulatory focus influenced their coping and resilience. It drew a contrast between a ‘promotion focus’, concerned with growth and progress, and a ‘prevention focus’, which is concerned with safety and responsibility: the former was found to enhance resilience and was associated with fewer symptoms of depression and anxiety than the latter.

## Aims

Little is known, therefore, about the lived experiences of Syrian refugees in the UK (and Western countries in general) in relation to resilience, what helps them to endure a long and traumatic journey to their chosen country of settlement and what helps them on a day-to-day basis to persevere and build a new life there. This study, addressing a significant gap in the literature, set out to explore factors that enhance the resilience of Syrian refugees in the UK.

## Method

### Sample

Purposive sampling^33^ was used to identify participants, making sure that they were as representative as practically possible of the Syrian community of refugees in the UK, in terms of gender and ethnic and religious backgrounds. This resulted in eight Syrian refugees (three women and five men) being interviewed. This is in accordance with the literature on interpretative phenomenological analysis (IPA), which recommends between six and eight participants in order to maintain the idiographic focus of IPA.^34^ The participants were all adults who had arrived in the UK as a result of the conflict in Syria between 6 and 12 months prior to the interview date, they were all registered with a general practitioner, had a place to live and were sufficiently grounded in their experience to consent to taking part in the study. The mean age of the participants was 34 years, ranging from mid-20s to mid-40s; all of them were married and all but one had children.

### Data

The interviews took place face to face, in Arabic, were audio-recorded and lasted between 39 and 73 min each. Participants were offered compensation for their travel expenses.

The interviews were semi-structured, using an interview schedule with questions focusing on the participant's resilience and coping in relation to the period prior to leaving Syria, their journey to the UK and their life since arriving in the UK. The interview schedule is available on request from the author and includes open-ended questions such as: ‘What in your view have been the important factors that have helped you cope with your difficulties?’ and ‘What helps you cope on a day-to-day basis now?’

It is worth mentioning that the word ‘resilience’ has no equivalent word in Arabic. The word *Muruna* (مرونة) has often been used to refer to resilience, but it means flexibility and adaptability without necessarily implying the presence of adversity. This created a challenge in conducting the study, which aimed to explore a concept that has no word to express it in the native language of the participants. When conducting the interviews, therefore, Arabic words that translate into coping, patience, perseverance and carrying on were used to refer to resilience.

### Analysis

Interpretative phenomenological analysis was considered the most appropriate method to address the research question. IPA is both a data analysis tool and a research methodology concerned with exploring people's lived experiences in relation to a particular phenomenon and in a particular context.^34^ IPA is also embedded in the hermeneutics, i.e. a phenomenon can only be understood by being subjected to interpretation.^35^ The researcher is therefore not a neutral investigator extracting ‘truths’ from the data. Instead, he or she is actively engaged in a process of interpreting the participant's experience in order to understand it, adding another layer of depth, sophistication and meaning to the world-view presented by the participant.^36^

IPA is increasingly being used in psychology and health research and is thought to be particularly useful when researching marginalised or disadvantaged groups such as refugees,^37^ as it is consistent with strength-based approaches as opposed to illness models or deficit approaches.^38^ A number of researchers have indeed already used IPA in exploring refugees’ experiences.^30,39^

Data analysis in IPA involves three levels of exploratory comments on the transcript:^34^ descriptive, taking what the participant says on face value; linguistic, commenting on the participant's use of language, such as the use of metaphors and culturally bound phrases; and conceptual, in which the researcher interrogates the data in light of their own experience.^34^ The themes emerging from this dynamic process are organised into superordinate categories, each of which includes a number of more detailed themes.

### Ethics

The study was approved by the Ethics Committee of the University of Chester. Before starting the interview, the participants read the participant information sheet and signed the consent form. The researcher also explicitly discussed with the participants the possible benefits and the potential harm to them as a result of taking part in the study and gave them a ‘Helpful Resources and Useful Information for Refugees’ leaflet. Moreover, the researcher's background, i.e. being Syrian and a psychotherapist, helped him to conduct the interviews in a supportive and sensitive, including culturally sensitive, manner that minimised the potential harm to the participants.

## Results

Following rigorous analysis of the data, three superordinate themes were arrived at as factors influencing participants’ resilience. Each of these major factors encompasses a number of detailed themes explaining, from the participants’ perspectives, how these factors helped their resilience. These themes are described below in order of how commonly they were reported by the participants.

### Interpersonal factors

This category of resilience factors was by far the most commonly cited by participants, all of whom clearly perceived their resilience as an interpersonal experience. They described the significant influence of their family and their connection with others on their ability to cope. This was expressed in various ways outlined in the themes below.

#### Direct support from family

Participants stated that they received emotional, practical and financial support from their families which helped them cope and carry on, as in this quote by Participant 1 describing the support he had received from his father:

‘I was putting all my weight on him, [… ] he was always supporting us and encouraging us to be patient’ (P1)

or in the following quote from Participant 8 describing the effect of her parents’ support:

‘So they helped me forget what I was going through, especially that I was newly-wed’ (P8).

#### Responsibility towards the family

All participants stated that the responsibility they felt towards their family gave them a reason to carry on and enhanced their resilience, as summed up in Participant 3's words:

‘When I collapse, the rosary beads will fall and people I love will also collapse’ (P3).

Participant 3 describes how her new-born baby, who starts off as a source of stress and despair, becomes her source of strength:

‘I'd look at him [the baby] and think: why did I bring you to life now? But then I think, no, he would give me strength, I felt he helped me be strong’ (P3).

Another participant referred to his journey across the border:

‘When I crossed the Turkish border I felt as if my children were pushing me, as if to say ‘keep going!’’(P5).

Furthermore, this responsibility towards their families continues to motivate Syrian refugees in building a life for themselves and their families in the UK:

‘What helps me cope here is only one thing: that I want to provide good education for my children’ (P1).

Participant 1, quoted above, is a young man whose successful business was completely ruined as a result of the war in Syria. He refers to how his children have become his motivating factor after he moved to the UK:

‘It is very unlikely that I would ever go back to innovation and to continue what I had started. The circumstances that I had before have changed, so I can only invest in my children now’ (P1).

#### The sense of community

The fact that refugees shared the suffering and had a common goal also helped them to persevere and push through the pain:

‘You see someone robust like a mountain, a woman whose three children have been killed and she says “Thank God, it is all for God's sake or for the sake of the oppressed and for justice”’ (P2).

Furthermore, participants compared the level of suffering they endured with that experienced by others who are worse off:

‘What helps me to not give up is that I say: when one sees other people's suffering he feels better about his own’ (P4).

### Factors related to religion, faith or belief systems

Although one participant (P5) did not feel that his religion or faith had a part to play in his resilience, the study showed clearly that faith and religion played a significant role in helping participants cope. There were individual differences in the way this role manifested itself, however, as illustrated below.

#### Fate

Participants derived great comfort and security from the conviction that God has already decided their future, is aware of their circumstances and has His own providence in what is happening. This belief helped them accept and be prepared for what might come their way, as is the case for Participant 1, who crossed the Mediterranean Sea, fully aware of the risk involved in this journey:

‘If I'm destined to live, then I won't die. I might come very close to death but I won't die’ (P1).

Another participant referred to the relief that acceptance of God's fate gives her:

‘Oh God! I accept your fate and judgement whatever happens. If you accept your situation and are content, this makes you feel comfortable’ (P3).

#### Religion providing hope and protecting against suicide

Participants relied on their faith to derive contentment, using religious texts to remain grounded:

‘Truly with hardship comes ease’ (P8, Quranic verse).

Participants also felt that their faith stopped them from taking their own lives, as implied in this quote:

‘Sometimes I think of killing myself because of how depressed I feel. It is too much! Seriously, how could a non-believer survive in these circumstances?’ (P8).

#### Religion providing meaning

Faith seemed to inform the values and philosophy of life of the participants, providing meaning and context to their suffering and helping them make sense of it.

For Participant 7, for example,

‘There is providence behind what is happening’ (P7).

Participant 8 goes further to reframe her suffering in the context of what she sees as God's intentions, therefore turning a desperate situation into a positive one:

‘ … it is one of two things: it's either a test from God, because He loves us, or in order for Him to forgive our sins. In both cases it's positive’ (P8).

#### Protection and help from God

Some participants believe that God actually intervenes to help them at times of need and hardship, including during the journey in the sea:

‘When I saw it with my own eyes I realised that the chance of surviving would be no higher than 15%. But people are managing to cross the sea not with their own effort, but with God's help’ (P1).

### Personal factors

These factors concern personal values, attributes, attitudes and coping styles that are unique to the participants’ themselves, who found that they helped them cope and remain resilient.

#### Hope and optimism

Participants held on to a hopeful and optimistic outlook, which helped them to accept their reality and keep going, as expressed by two female participants in beautiful metaphorical language:

‘ … we always look at the full half of the glass. When one looks at the full half of the glass one feels content’ (P8)

‘When I see a dead flower, I don't want to see that it is dead; I want to see it as a flower still’ (P3).

#### Looking ahead

Linked to hope and optimism was the idea that participants had their eyes on the future, with clear goals for themselves and their children, as detailed in this quote by a young participant who, after arriving to the UK, said he was looking at the next step of his journey:

‘Thank God I arrived. And now what should I do? I need to work on getting my family over, then find a job, learn the language and get integrated into society’ (P4).

#### Moral values

Participants referred to having moral values that kept them going and grounded them in what matters to them, such as speaking truth to power:

‘To be able to speak your mind among people who are all not telling the truth, that in itself is a mission’ (P7);

or having a clear conscience:

‘The important thing is that I know that my conscience is clear, that my hands are not stained with blood, that my parents are pleased with me that I did not join any of the fighting groups’ (P4).

#### Coping skills and defences

Unsurprisingly, participants described various coping skills and defence mechanisms that they developed to help themselves cope with extreme suffering. These strategies and defences, and the way they were expressed, varied from one participant to another, and included denial and dissociation:

‘We had to “beautify reality” in order to convince ourselves that there was no trouble in the country. The country was being destroyed. We'd dissociated from reality for some time’ (P3);

or keeping a daily routine:

‘I started having a habit of waking up and having a cup of coffee or tea, I got used to this routine. It improved my mood’ (P1).

## Discussion

The study showed that, for the Syrian refugees who were interviewed, resilience was enhanced by three groups of factors: interpersonal factors; factors related to religion, faith or belief systems; and personal factors. These findings are consistent with the literature on resilience among refugees from other countries, such as Schweitzer et al^30^ on Sudanese refugees, Sossou et al^39^ on Bosnian women refugees and Thomas et al^8^ on urban refugees in Nepal.

In the Syrian context, Arenliu et al^40^ interviewed 30 Syrian refugee families in Istanbul, Turkey, asking them about stressors and coping. Though grouped differently, the findings of his study were broadly similar to the findings of the current study, and emphasised the importance of support from family members, faith and a belief system (such as using prayers and the belief in the will of God), being positive and optimistic and looking ahead to a better future in the new country.

The significance of interpersonal and family factors, emphasised by all the participants, suggests that refugees saw their resilience as an interpersonal, interactive and dynamic process: they derived great support from their family but they also felt they had a responsibility towards their family to stay strong and cope well.

The collective nature of refugees’ experience, i.e. the fact that many people are in the same situation, is a defining feature of their suffering and seems to play a positive role in helping them cope as they realise they are not alone and will always find others who are in a worse position than their own (social comparison theory^41^). Some may argue that, as a result, refugees may end up minimising their own suffering as they compare it with that of other fellow refugees, a cognitive bias referred to ‘downward comparisons’,^27^ and some may wonder whether this constitutes a form of denial. This is a valid argument but one that is beyond the scope of this paper. Suffice it to say that this seems to be an adaptive way that refugees have found helpful, as it helped them acquire a perspective on their situation that is less self-centred and more outward-looking and compassionate towards others, and it should therefore be validated and not pathologised.

The role of religion and faith in enhancing resilience was expressed by the participants in different ways, such as the belief in fate, the sense that faith protects from suicide and provides hope, purpose and meaning to life and suffering, as well as the belief in direct protection from God. This is also in keeping with studies carried out among other refugee groups, such as Overland & Yenn^42^ on Khmer refugees in Norway, and Kashyap & Sharma^28^ on Tibetan refugees. This suggests that refugees may cope better if their hardship is given a meaning and a context, which religion often provides.^29^ Importantly, the role of religion as a buffer against suicide, as suggested in this study, was also shown in previous studies, such as Thomas et al's^8^ study on urban refugees in Nepal. In this context, it has been suggested^40^ that people may actively turn to religion and faith when in extreme situations, to regain some certainty and control. The potential impact of war on people's religiosity and faith is therefore worth studying further, including the possibility that people may feel let down or betrayed by a God in whom they had put their trust.

The study participants identified various personal attributes, attitudes and coping styles as being related to their ability to cope. These included hope and optimism, looking ahead, having moral values and possessing certain coping skills. This seemingly heterogeneous group of factors, referred to sometimes as ‘internal resources’,^6^ has also been highlighted in previous studies on refugees.^13,43,44^ It may be argued that these personal attributes define in essence what resilience is and how it manifests in ‘resilient people’. This argument is linked to the debate on whether resilience is a personality trait^23^ or a dynamic process denoting the quality of the interaction between the individual and their environment and circumstances. Further exploration on this subject is beyond the scope of this paper.

### Limitations

Although it provides great insight into the refugees’ lived experience of resilience and coping, the study has several limitations. As mentioned above, the interviews were carried out in Arabic, which has no word for ‘resilience’. The researcher did his best to communicate the concept clearly to the participants, but it may be possible that the researcher and the participants did not share exactly the same understanding of what is being asked. A close look at the interview transcripts, however, suggests that the participants did address resilience in their answers.

Furthermore, conducting the interviews in the participants’ own language facilitated their self-expression and enhanced rapport, leading to more meaningful and accurate answers. The possibility that some concepts may have been lost in translation, however, cannot be ruled out completely, especially that the researcher translated the transcripts into English himself, with a potential for bias.

Moreover, the study set out to address resilience factors among refugees, rather than assessing whether or not they saw themselves as resilient. This therefore raises the possibility that the participants may have felt that they had to come up with resilience factors even when they did not feel that they were coping well.

The sample size (eight participants) is in keeping with IPA methodology, and attempts were made to make the sample as representative as possible, in terms of gender and ethnic and religious backgrounds. However, the sample cannot be claimed to represent all Syrian refugees, especially in other countries or those who have been settled for a long time as opposed to the 6–12 month cut-off used in this study. Nevertheless, as is the case with qualitative research, the aim of the study is not to achieve generalisable results but to provide an in-depth account of a phenomenon as experienced by the participants.

Finally, all participants had been granted asylum at the time of the interview and they may have been in ‘the honeymoon period’,^45^ a term that denotes a sense of safety and relief following extreme stress or trauma. This, along with the retrospective nature of the reporting, might have influenced the way the participants perceived and communicated their sense of resilience and might therefore have affected the results of the study, with participants expressing their present state of mind and current beliefs. This type of bias is inevitable in this kind of research,^30^ which is more interested in the participants’ lived experiences than in objective reporting.

### Implications

The study findings call for a new way of addressing refugees, by researchers and workers in the field, organisations, policy makers and society at large, as resilient individuals with strengths and adaptive qualities as opposed to victims or passive recipients of state support.

In terms of working therapeutically with refugees, this study encourages therapists to see themselves as facilitators of refugees’ journeys of recovery as opposed to saviours of helpless victims. It also advocates that therapists help refugees explore and build on their strengths and not simply treat their psychopathology and vulnerabilities. Furthermore, the study shows that resilience is essentially an interpersonal process and that family plays a great role in resilience. This suggests that more systemic and relational approaches to therapy, such as group and family therapy, should be considered in working with refugee populations who require professional psychosocial support.

The great significance that participants assign to their faith or religion necessitates that therapists and workers in the field engage with refugees on the subject and do not shy away from it, and that they possess a level of ‘spiritual competence’,^46^ i.e. familiarity with the religious and spiritual beliefs and practices of their patients or clients and the subtlety and diversity in the ways people relate to or express their religion and belief system. Training and supervision are also paramount in ensuring that professionals are skilled and comfortable in exploring these topics with refugees.

Finally, the study participants stressed the importance of family support. Yet, most of them arrived in the UK without their families, who were left in Syria or in neighbouring countries for prolonged and uncertain periods. Policy makers, the Home Office and refugee support services should therefore focus on speeding up the processing of asylum applications and facilitating the family reunion process.

## Data Availability

The data that support the findings of this study are available from the corresponding author on reasonable request.

## About the author

**Mustafa Alachkar**, MRCPsych, MSc, is a consultant psychiatrist and medical psychotherapist of Syrian origin, currently working in Pennine Care NHS Foundation Trust, Ashton-under-Lyne, UK.
